# Photodynamic therapy changes tumour immunogenicity and promotes immune-checkpoint blockade response, particularly when combined with micromechanical priming

**DOI:** 10.1038/s41598-023-38862-8

**Published:** 2023-07-19

**Authors:** Catarina S. Lobo, Maria Inês P. Mendes, Diogo A. Pereira, Lígia C. Gomes-da-Silva, Luis G. Arnaut

**Affiliations:** grid.8051.c 0000 0000 9511 4342 CQC, Chemistry Department, University of Coimbra, 3004-535 Coimbra, Portugal

**Keywords:** Immunology, Oncology, Lasers, LEDs and light sources, Cancer, Cancer imaging, Cancer microenvironment, Cancer therapy, Tumour immunology, Cancer

## Abstract

Photodynamic therapy (PDT) with redaporfin stimulates colon carcinoma (CT26), breast (4T1) and melanoma (B16F10) cells to display high levels of CD80 molecules on their surfaces. CD80 overexpression amplifies immunogenicity because it increases same cell (*cis*) CD80:PD-L1 interactions, which (i) disrupt binding of T-cells PD-1 inhibitory receptors with their ligands (PD-L1) in tumour cells, and (ii) inhibit CTLA-4 inhibitory receptors binding to CD80 in tumour cells. In some cancer cells, redaporfin-PDT also increases CTLA-4 and PD-L1 expressions and virtuous combinations between PDT and immune-checkpoint blockers (ICB) depend on CD80/PD-L1 or CD80/CTLA-4 tumour overexpression ratios post-PDT. This was confirmed using anti-CTLA-4 + PDT combinations to increase survival of mice bearing CT26 tumours, and to regress lung metastases observed with bioluminescence in mice with orthotopic 4T1 tumours. However, the primary 4T1 responded poorly to treatments. Photoacoustic imaging revealed low infiltration of redaporfin in the tumour. Priming the primary tumour with high-intensity (~ 60 bar) photoacoustic waves generated with nanosecond-pulsed lasers and light-to-pressure transducers improved the response of 4T1 tumours to PDT. Penetration-resistant tumours require a combination of approaches to respond to treatments: tumour priming to facilitate drug infiltration, PDT for a strong local effect and a change in immunogenicity, and immunotherapy for a systemic effect.

## Introduction

Photodynamic therapy (PDT) is a minimally-invasive therapeutic procedure that can be curative in early-stage solid tumours^1^. The procedure involves the administration of a photosensitizer that absorbs red or near-infrared light, and when it is electronically excited in the presence of molecular oxygen, it generates reactive oxygen species (ROS) that are cytotoxic and destroy the illuminated tissues. Although PDT activates cell death mechanisms that stimulate the immune system and can have systemic effects^2,3^, these are generally insufficient to change the current paradigm that most cancer deaths are due to metastases resistant to conventional therapies^4^.

The active stimulation of the host immune system to mount an antitumour immune response capable of controlling tumour growth and dissemination has been pursued for a century, and recently met with considerable success. Immune-checkpoint blockade (ICB) therapy, which employs monoclonal antibodies (mAb) to block inhibitory immunoreceptors (checkpoints) and unlock the antitumour function of immune cells present in the immunosuppressive tumour microenvironment (TME), revolutionized oncology and reached the status of first-line treatment against various cancers^5–7^. ICB therapies recover the cytotoxic potential of CD8+ T cells with antigen receptors (T-cell receptors, TCRs) that recognize tumour-derived peptides bound to major histocompatibility complex (MHC) molecules on tumour cells and enable cytotoxic T cells to eliminate tumour cells anywhere in the organism. However, although nearly half of US cancer patients are eligible for ICB therapies, only 13% of them respond to such therapies^8^.

Standard chemotherapy^9^, radiotherapy^10^ or photodynamic therapy (PDT)^11^ can induce immunogenic tumour-cell death that releases damage-associated molecular patterns capable of dendritic cell maturation and recruitment, and activation of cytotoxic T cells^12^. Combinations of these therapies with ICB therapies raised new hopes to achieve higher cure rates in metastatic cancer. ICB + PDT combinations are particularly interesting because PDT is minimally invasive, has negligible systemic effects, lacks resistance mechanisms, preserves organ functionality, changes the context of immune checkpoint expression and disrupts the TME in a manner that releases an array of tumour-specific antigens^1,3,13–15^. The drug-to-light interval (DLI) between the administration of the photosensitizer and illumination of the tumour determines the primary target of the oxidative stress, which may be the vasculature (vascular-PDT) for DLI < 60 min or the cells (cellular-PDT) for DLI > 12 h. These regimes lead to different biological responses with distinct immunological components^16^, and allow for spatiotemporal control of the therapy.

Here, we report PDT of mice with syngeneic tumours using redaporfin, a photosensitizer in Phase II clinical trials of advanced head and neck cancer^17^, in combination with ICBs. Our interest in redaporfin-PDT comes from its high cure rates in a variety of tumours^16,18–20^, dependence of cure rates on cytotoxic CD8+ T cells^21^, damage to the endoplasmic reticulum and Golgi apparatus capable to set a hierarchically organized cascade of signaling events to stimulate classical apoptosis^22^, and ability to trigger immunogenic cell death and generate prophylactic vaccines^23^. We studied BALB/c mice with CT26 (colon carcinoma), C57BL/6 mice with B16F10 (melanoma) and BALB/c mice with orthotopic 4T1 (mammary) tumours, to cover a wide range of tumour immunogenicities. CT26 responds to chemotherapies similarly to human colon cancer^24^, and shows high immunogenicity and responsiveness to ICB^25^. B16F10 spontaneously forms metastases post-implantation into mice^26^, and does not respond to ICB^27^. 4T1 mimics the stage IV human breast cancer and is immunosuppressive and highly aggressive, capable of metastatic spread in mice^28,29^. We investigated changes in the expression of CD80, CTLA-4 and PD-L1 molecules on tumour cells to understand their response to ICB + PDT. This was complemented by bioluminescence imaging of metastases and by photoacoustic tomography (PAT) of tumours with real-time imaging of drug and oxygen distribution. Challenged by the of lack of response of orthotopic 4T1 tumours, we introduced tumour priming with pressure pulses to enhance the exposure of cancer cells to therapeutics. Our findings reveal the roles of immunogenicity and TME drug infiltration in treatment outcomes.

## Results

### Activation and regulation of T cell responses

**Figure 1:**
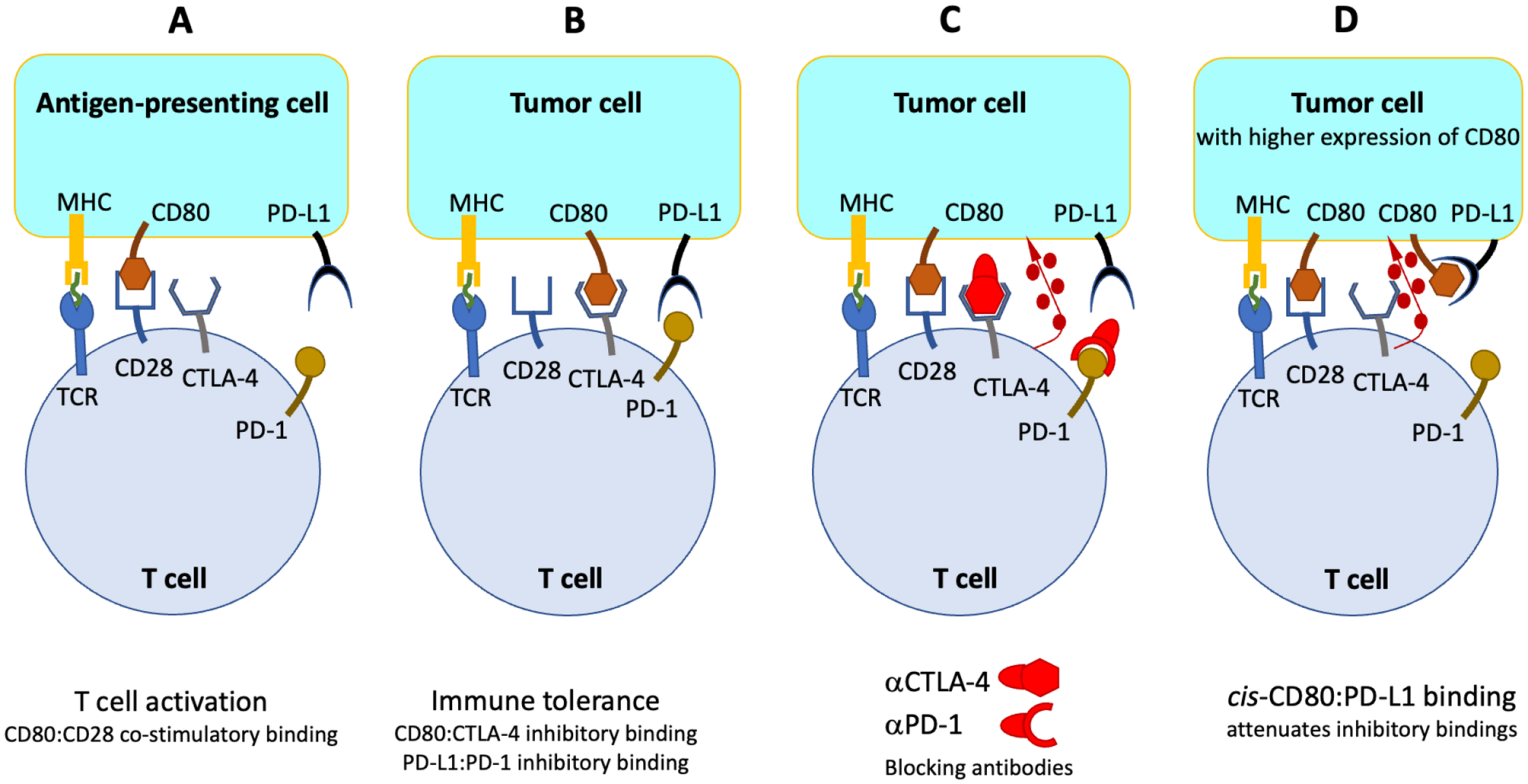
Activation and regulation of T cell responses. (**A**) T cell activation with presentation of an antigen (in green) by the MHC of an antigen-presenting cell to the cognate TCR of a naive T cell, together with co-stimulatory binding between a CD28 receptor and CD80. (**B**) Immune tolerance induced by binding of CTLA-4 and/or PD-1 in T cells to their ligands in tumour cells. (**C**) ICB therapy where CTLA-4-blocking antibodies (αCTLA-4), and/or PD-1-blocking antibodies (αPD-1), promote antibody-dependent cytotoxicity represented by the release of cytolytic mediators (dark red circles). (**D**) Overexpression of CD80 molecules on tumour cells enables *cis*-CD80:PD-L1 interactions that attenuate PD-1:PD-L1 binding and inhibit CTLA-4:CD80 binding, thus allowing for the release of cytolytic mediators. Although CD80 is represented at the surface of tumour cells to illustrate the various interactions, CD80 is not expressed at the surface of most tumour cells.

T cell activation demands both MHC-peptide-TCR engagement and co-stimulatory binding between CD28 receptors expressed on CD8+ T cells with CD80 (i.e., B7.1) or CD86 (i.e., B7.2) molecules on antigen presenting cells^30^, Fig. 1A. CD28:CD80 interactions reduce the number of TCRs that must be triggered for T cell activation and enhance cell proliferation. Response to pathogenic infection and self-tolerance are balanced by various stimulatory and inhibitory signals, which control T cell mediated immunity. Most ICB therapies target the T cells inhibitory receptors CTLA-4 (cytotoxic T lymphocyte antigen-4)^31^ and PD-1 (programmed death-1)^32^. CTLA-4 receptors are expressed on activated CD4+ and CD8+ T cells and on Treg cells, as well as on tumour cells themselves^33,34^. They primarily regulate the amplitude of the early stages of T cell activation. CTLA-4 binds CD80 molecules with much higher affinity than CD28, which impedes CD80 to stimulate CD28 and prevent T cell anergy during the contact of T cells with antigen-presenting cells^31^. PD-1 receptors are highly expressed in tumour-infiltrating lymphocytes^35^. PD-1 binding to its ligands PD-L1 or PD-L2 limits the activity of T cells in peripheral tissues and controls autoimmunity. PD-L1 expressed on the surface of tumour cells acts as a molecular shield to prevent cytolysis mediated by T cells because PD-1:PD-L1 interaction antagonizes CD28:CD80 co-stimulation^36^. Intrinsically higher PD-L1 expression in tumours is associated with T cell anergy. Improved response rates to PD-L1 and PD-1 blockade are observed in patients with cells expressing high PD-L1 levels in the TME, although this is less evident when PD-L1 is expressed by the tumour cells^37^. Immune tolerance induced by the binding of CTLA-4 and PD-1 to their ligands eventually leads to tumour cell evasion of immunosurveillance, Fig. 1B. mAbs targeting T cells inhibitory receptors (αCTLA-4 and αPD-1) are illustrated in Fig. 1C.

The original view that CTLA-4:CD80 and PD-1:PD-L1 binding between TCRs and ligands in other cells (*trans* binding) are at the essence of tumour immune evasion, was recently enriched with the knowledge that PD-L1 also binds to CD80 when these molecules are overexpressed in the same cell (*cis*-CD80:PD-L1) but not when they are in different cells (no *trans*-CD80:PD-L1)^38^. *cis*-CD80:PD-L1 disrupts PD-1:PD-L1 binding^39^ but does not affect CD28:CD80 binding and signalling^40,41^. Overexpression of CD80 on tumour cells expressing PD-L1 can attenuate the binding of PD-1 to tumours^42^, and abrogation of PD-1 function by *cis*-CD80:PD-L1 associations was proposed as a universal strategy to overcome PD-1 induced immune tolerance^39^. *cis*-CD80:PD-L1 interaction also inhibits CTLA-4:CD80 binding by an avidity effect rather than by directly blocking the CTLA-4 binding site on CD80^32,40^, Fig. 1D. Although c*is*-CD80:PD-L1 disrupts inhibitory functions of both CTLA-4 and PD-1, tumour cells often have low CD80 expression and this mechanism is not effective^41^.

### Redaporfin-PDT increases CD80 expression in tumour cells and alters the expression of immune molecules

**Figure 2:**
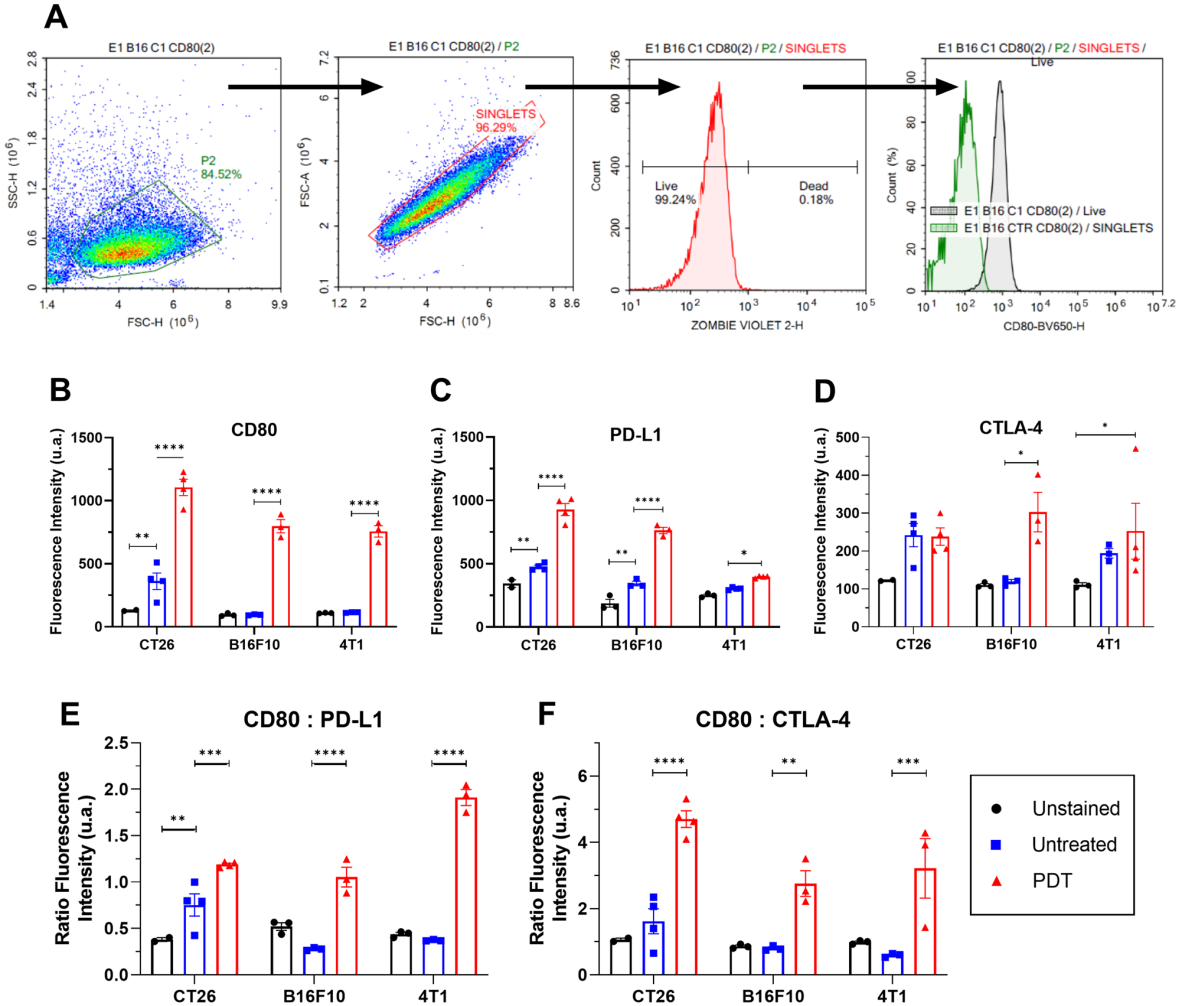
Expression of immune molecules by tumour cells in vitro. (**A**) Gating strategy used in flow cytometry to evaluate changes in expression of immune molecules triggered by redaporfin-PDT: the cell population was selected from the SSC/FSC plot, followed by a gate for the singlet events, rejection of death cells marked positive in the zombie violet assay, measurement of the mean fluorescence of the dye corresponding to each molecule (CD80, PD-L1 and CTLA-4), and comparison with the untreated and unstained samples. Expression of (**B**) CD80, (**C**) PD-L1 and (**D**) CTLA-4 molecules on the three cell lines on unstained cells, untreated cells (light only) and after PDT. (**E**) CD80/PD-L1 ratio. (**F**) CD80/CTLA-4 ratio. *p*-values < 0.05 were considered significant with (*), *p*-values < 0.01 (**), *p*-values < 0.001 (***), *p*-values < 0.0001 (****).

**Figure 3:**
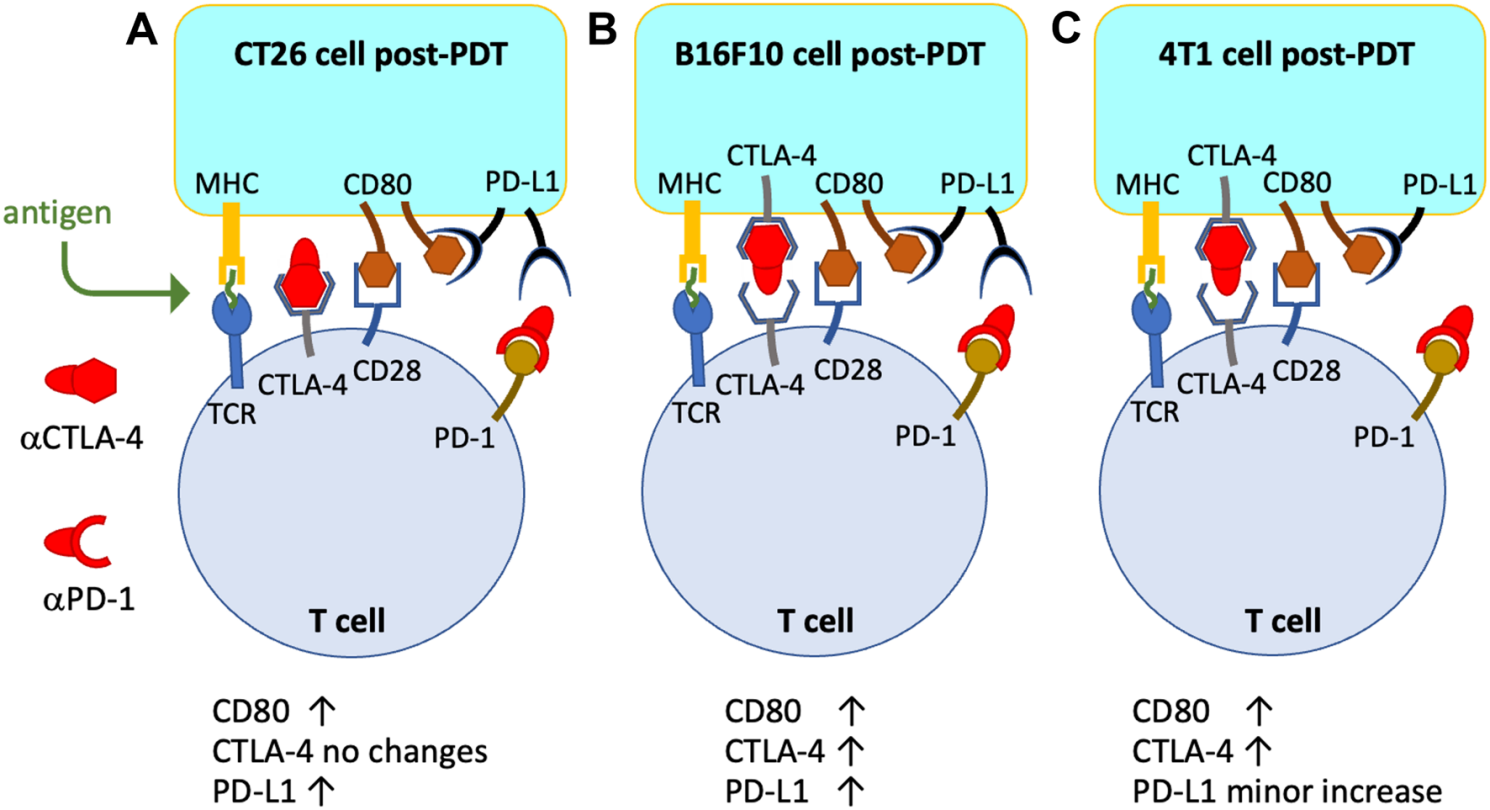
Expression of CD80, PD-L1 and CTLA-4 molecules on the surface of CT26, B16F10 and 4T1 cells after PDT. (**A**) CT26 cells illustrating the increase in CD80 and PD-L1 expressions and consequent *cis*-CD80:PD-L1 biding; the absence of change in CTLA-4 suggests virtuous combinations with αCTLA-4. (**B**) B16F10 cells illustrating the increase in CD80, PD-L1 and CTLA-4 expressions; increase of PD-L1 and CTLA-4 may contribute to the exhaustion of mAbs. (**C**) 4T1 cells illustrating the increase in CD80 and CTLA-4 expressions, but keeping the expression PD-L1 almost unchanged; *cis*-CD80:PD-L1 binding in these tumour cells may disrupt PD-1:PD-L1 binding and inhibit *trans*-CTLA-4:CD80 binding.

With few exceptions, notably CT26 cells^43^, tumour cells do not express CD80 molecules on their surface and hence are largely invisible to the immune system^30^. Our flow cytometry data (Fig. 2A) confirms these observations. However, PDT dramatically increases CD80 expression (Fig. 2B). The increased expression of CD80 may enable *cis*-CD80:PD-L1 binding, which disrupts PD-1:PD-L1 binding and inhibits CTLA-4:CD80 binding. This should be particularly advantageous when the CTLA-4 expression is not affected by PDT (Fig. 3A).

PDT increases the expression of PD-L1 by CT26 and B16F10 cells, and marginally also by 4T1 cells (Fig. 2C). Higher PD-L1 expressions are usually associated with better αPD-L1 responses. However, we are observing this higher expression after PDT, which suggests the need for more αPD-L1 to attain the same blockade level. This may be compensated by the increased CD80 expression, which will lead to more *cis*-CD80:PD-L1. 4T1 cells have the highest CD80 to PD-L1 ratio after PDT.

The surfaces of tumour cells exhibit low CTLA-4 expression, but PDT increases CTLA-4 expressions in B16F10 and 4T1 cells (Fig. 2D). This may demand more αCTLA-4 to attain an equivalent blockade (Fig. 3B). Given that *cis*-CD80:PD-L1 negatively affects the CTLA-4:CD80 interaction^40^, the unchanged CTLA-4 levels of CT26 tumours after PDT suggest that αCTLA-4 + PDT combinations may take advantage from the increase in the expression of CD80 and PD-L1 in this cell line.

The various changes in the expressions of CD80, PD-L1 and CTLA-4 after PDT suggest four different cases: (i) increase of CD80 and PD-L1 expressions in CT26 and B16F10 cells may promote PD-1:PD-L1 and *cis*-CD80:PD-L1 interactions that compensate for each other, and αPD-L1 + PDT combinations may not offer therapeutic benefit in CT26 and B16F10 tumours; (ii) increase of CTLA-4 in B16F10 cells, in addition to CD80 and PD-L1, may reduce the therapeutic value αCTLA-4 + PDT in B16F10; (iii) CT26 cells do not increase CTLA-4 after PDT but increase CD80, and αCTLA-4 may benefit from combination with PDT in CT26 tumours; (iv) 4T1 cells increase CD80 more than PD-L1 or CTLA-4, which should increase *cis*-CD80:PD-L1 and disrupt both PD-1:PD-L1 and CTLA-4:CD80 binding, suggesting promising αCTLA-4 + PDT and αPD-L1 + PDT combinations in 4T1 tumours (Fig. 3C).

### Photoacoustic tomography and redaporfin tumour infiltration

3D images of redaporfin, oxy- and deoxy-haemoglobin in CT26, B16F10 and 4T1 tumours at 0, 0.25, 0.5, 1, 24, 48 and 72 h post-administration of redaporfin were obtained scanning each tumour at 6 wavelengths with a 0.5 mm step size. We ensured that scanning did not bleach redaporfin appreciable, by recovering its photoacoustic spectrum from the last scan in CT26 tumours (Supplementary Fig. S1). Supplementary video SV1 presents the first 4 min after redaporfin administration in a CT26 tumour. PAT offers a good measure of redaporfin distribution within the tumour although the quality of the images obtained for B16F10 tumours is limited by the absorption of melanin.

**Figure 4:**
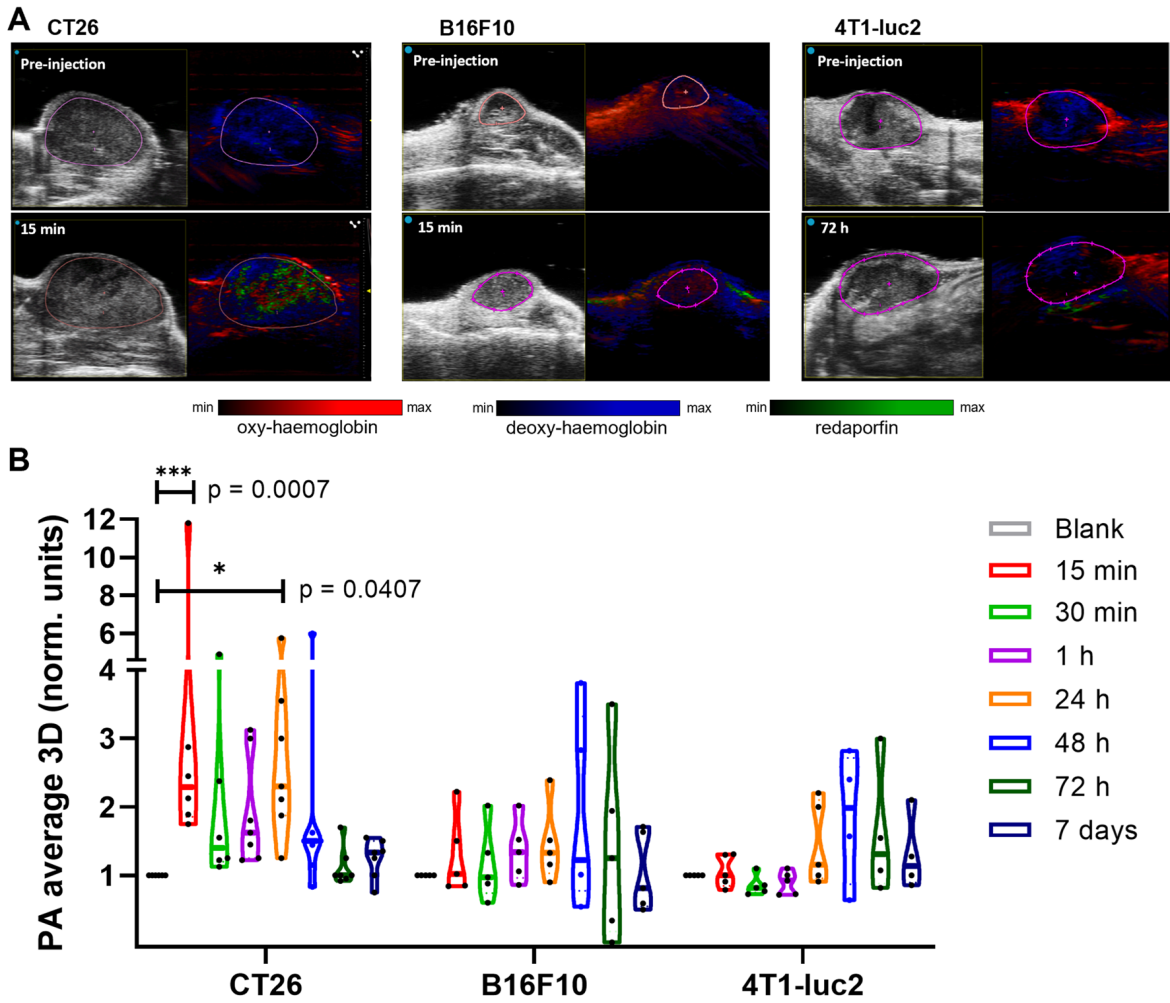
Redaporfin levels in tumours by PAT after i.v. injection of 1.65 ± 0.15 mg/kg. (**A**) Representative B-mode and photoacoustic unmixed images of CT26, B16F10 and 4T1-luc2 tumours before redaporfin i.v. administration and at the times indicated in the plots (see Supplementary videos SV2-4 for whole tumour imaging). (**B**) Violin plot representation of redaporfin tumour content at several timepoints; a blank initial acquisition before injection was performed and the following acquisitions were normalized to the blank. The dash represents the median of the data, and the dots represent the quartiles limits. Red: oxy-haemoglobin; blue: deoxy-haemoglobin; green: redaporfin.

Figure 4A shows the distribution of redaporfin, oxy- and deoxy-haemoglobin in the central plane of the tumours 15 min and 72 h post-administration. The amount of redaporfin in the whole tumour region of 4–6 animals at each timepoint was quantified and is displayed in the form of a violin plot in Fig. 4B. CT26 tumours accumulated the largest amount of redaporfin and 4T1 tumours the lowest. The signal from redaporfin in 4T1 tumours comes mostly from their periphery.

The amount of redaporfin inside a tumour is expected to correlate with its response to PDT. Indeed, systematic treatments of subcutaneous CT26 tumours in mice showed that vascular protocols (0.75 mg/kg redaporfin, 50 mJ/cm^2^ light at 749 nm, DLI = 15 min), which correspond to the largest amounts of redaporfin in the tumours (Fig. 4B), yielded the best cure rates (~ 85% for tumours with diameter $\phi$ = 5 mm)^19^. Although the dispersion of redaporfin distributions in subcutaneous B16F10 tumours did not allow for statistically significant differences, DLI = 15 min offered ~ 20% cure rates for 1.5 mg/kg and 105 J/cm^2^^18^. The distribution of redaporfin in orthotopic 4T1 tumours suggests that longer DLIs should be preferred for the treatment of these tumours. Hence, we selected DLI = 15 min for treatments of subcutaneous CT26 and B16F10 tumours, and DLI = 72 h for the treatment of orthotopic 4T1 tumours. In order to assess more readily the effect of the combination with ICB therapies, the redaporfin doses were lower, or the tumours were larger, than in the optimized redaporfin protocols^18,19^.

The 3D data collected additionally allows for the analysis of oxy- and deoxy-haemoglobin in the tumours, as shown in Fig. 6A, which are related to tumour oxygenation. Strikingly, the signal inside of 4T1-luc2 tumours is clearly dominated by deoxy-haemoglobin.

### Therapeutic benefits of αCTLA-4 administration in combination with redaporfin-PDT

**Figure 5:**
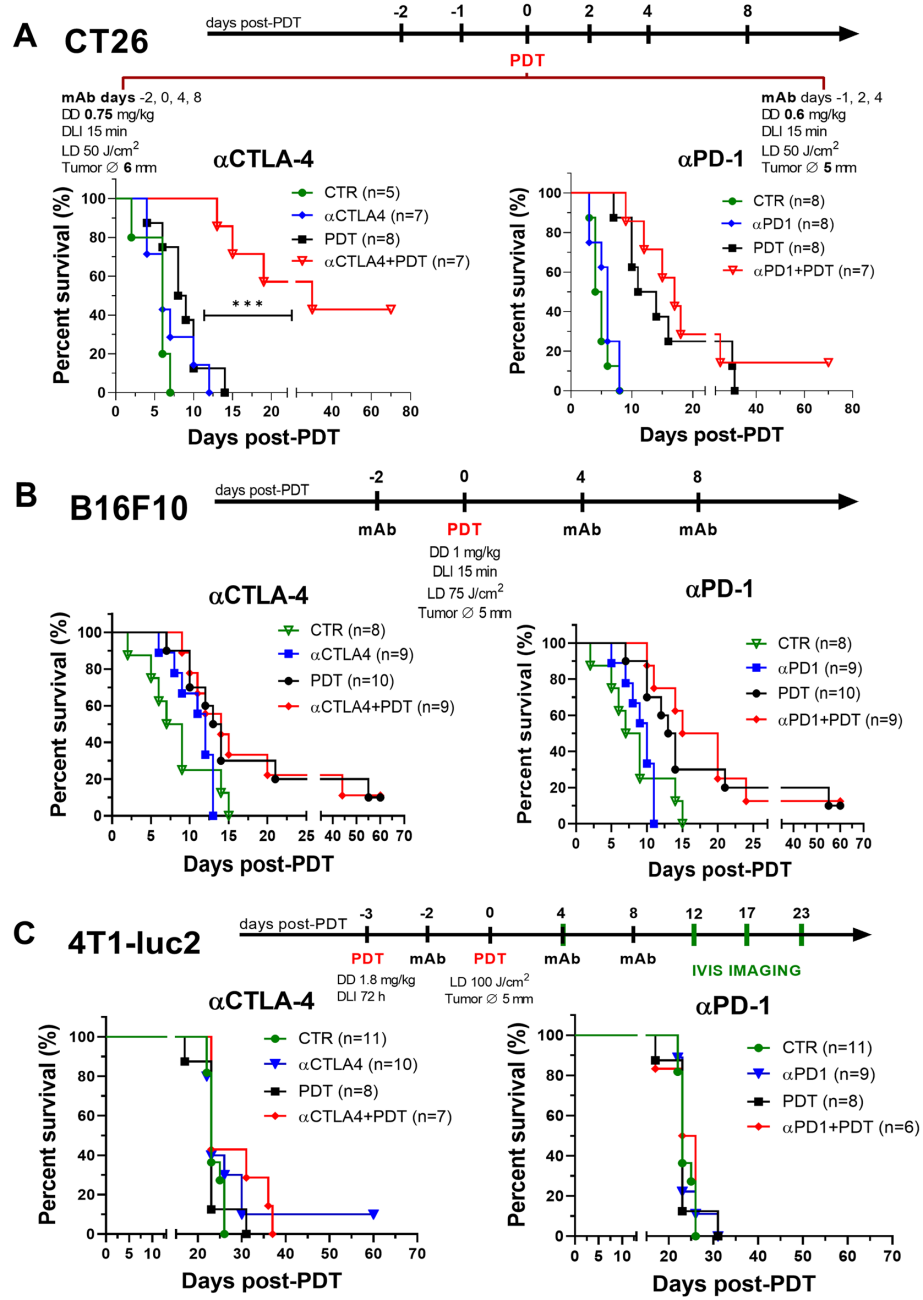
Kaplan–Meier curves representing % survival of mice in redaporfin-PDT combinations with CTLA-4 and PD-1 ICBs. (**A**) CT26, (**B**) B16F10 and (**C**) 4T1-luc2 tumour models. The protocol for antibodies administration is represented as a function of the PDT treatment day. mAb were administered 1 day before, 1 and 3 days after PDT or 2 days before, 30 min before, 4 and 8 days after PDT. CT26 tumours were treated with 0.6/0.75 mg/kg of redaporfin, DLI = 15 min, 50 J/cm^2^ @ 130 mW/cm^2^, 13 mm diameter frontal illumination. B16F10 tumours were treated with 1 mg/kg of redaporfin, DLI = 15 min, 75 J/cm^2^ @ 130 mW/cm^2^, 13 mm diameter frontal illumination. 4T1-luc2 tumours were treated with 1.8 mg/kg of redaporfin, DLI = 72 h, 100 J/cm^2^ @ 130 mW/cm^2^, 12 mm diameter transversal illumination. *DD* drug dose, *DLI* drug-to-light interval, *LD* light dose, *mAb* monoclonal antibodies, *Tumour ∅* tumour diameter.

Vascular-PDT of 5 mm CT26 tumours with 0.6 mg/kg redaporfin increased the median survival time of mice by 8 days with respect to control but was not curative (Fig. 5A). The schedule for the administration of αPD-1 did not improve survival over control. αPD-1 + PDT cured one animal but, for the size of our samples, did not offer a statistically-significant increase in survival. Vascular-PDT of 6 mm CT26 tumours with 0.75 mg/kg redaporfin increased the median survival of mice by only 2.5 days because tumours regrow in the periphery of illuminated area. The αCTLA-4 administration protocol did not improve survival but αCTLA-4 + PDT showed a remarkable improvement in median survival, by 24 days, and cured 43% of the mice. There is a clear benefit in the combination of redaporfin-PDT with αCTLA-4 (*p* = 0.003), as expected from the high expression of CD80 and low expression of CTLA-4 on the surface of CT26 cells after PDT.

Combinations of redaporfin-PDT with αPD-1 or αCTLA-4 did not increase cures rates of mice with B16F10 tumours beyond the 10% cure rate of redaporfin-PDT in the conditions employed in this study (Fig. 5B). The median survival time increased from 13.5 days with PDT to 17.5 days with αPD-1 + PDT but was not statistically significant. The kinetics of tumour growth were not significantly changed (Supplementary Fig. S2). Apparently, the higher expressions of PD-L1 and CTLA-4 by B16F10 cells after PDT require more mAbs to obtain a therapeutic response. Although we confirmed that B16F10 tumours do not respond to immunotherapies^44^, this is not translatable to human melanoma, which is a successful case of ICB therapy^45^.

Figure 5C shows that αPD-1 does not change the survival of mice with orthotopic 4T1 tumours and its combination with redaporfin-PDT is equally ineffective. αCTLA-4 cured one animal, but survival was not statistically significant. αCTLA-4 + PDT seemed to increase the median survival time of a subpopulation of the animals, but for the group sizes employed, it was not statistically significant. This is somewhat disappointing in view of the much larger expression CD80 than PD-L1 or CTLA-4 by 4T1 cells after PDT. The use of the 4T1-luc2 cell line allowed for the evaluation of metastases using their bioluminescence. The protocol for the implementation of 4T1-luc2 orthotopic tumours was designed to ensure that lung metastases would be observed even if complete tumour resection was performed on the day selected for treatment (Supplementary Table S1 and Figure S3).

### Lung metastases regress with αCTLA-4 combined with redaporfin-PDT

**Figure 6:**
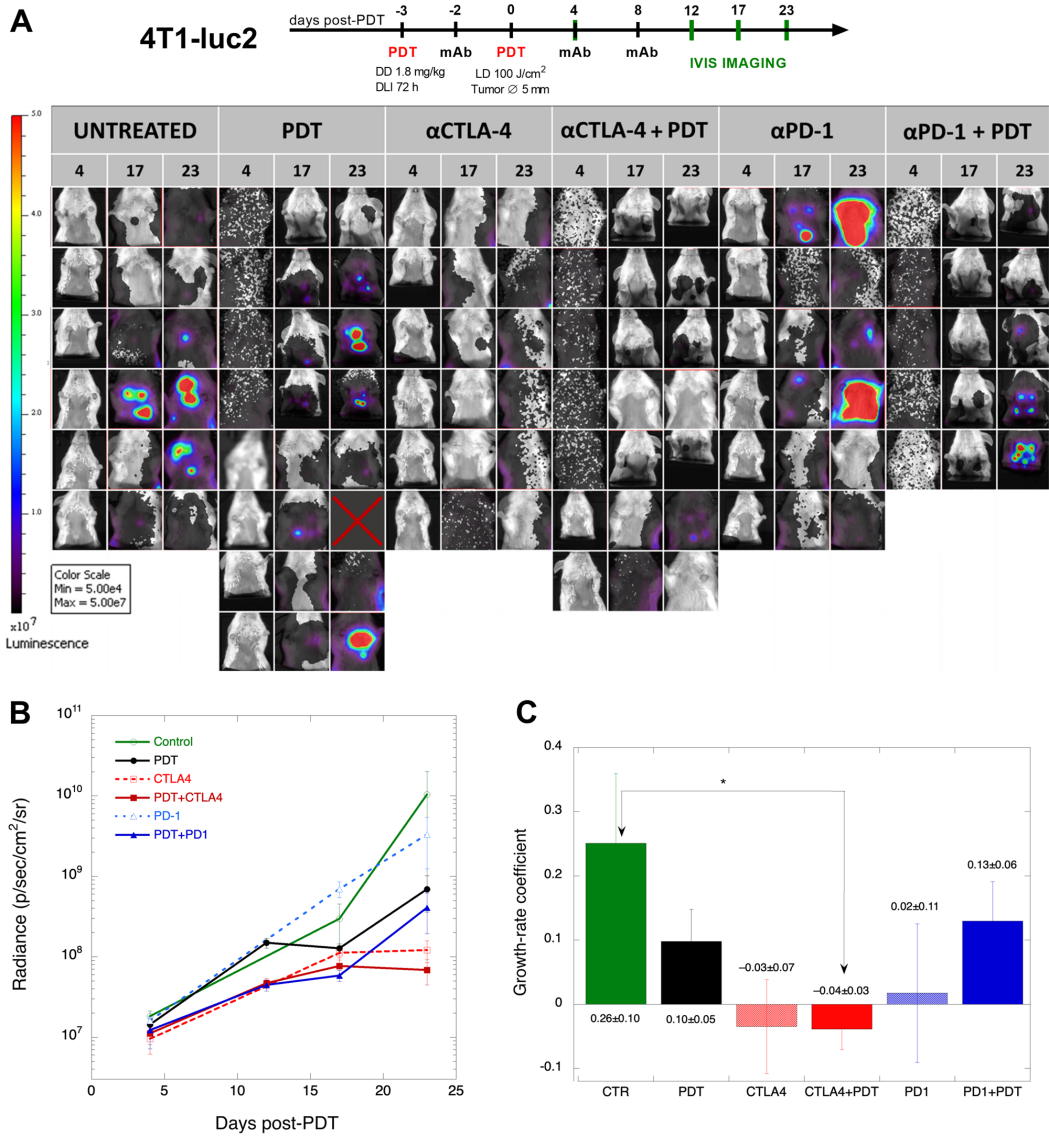
Lung metastases from orthotopic 4T1-luc2 tumours. (**A**) Bioluminescence of 4T1-luc2 metastases in the lungs collected 4, 17 or 23 days post-PDT; the primary tumour bioluminescence was blocked by an opaque cover but some background luminescent is still detected, which gives a dark grey colour (~ 10^7^ luminescence) rather than the white colour used to represent the absence of bioluminescence (~ 10^6^ luminescence); all images were taken in automatic mode and are presented in the same colour scale. (**B**) Average radiance and standard error of each group. (**C**) Growth rates of metastases from days 18 to 23 according to the Gompertz function, where negative rates represent tumour regression.

Figure 6A presents the bioluminescence images collected from the lungs of mice with orthotopic 4T1-luc2 tumours treated with PDT or immunotherapies. The first metastases were observed 20 to 25 days after the inoculation of 4T1-luc2 cells in the mammary fat pad (12 days post-PDT). Some animals treated with αPD-1 showed more metastases than the untreated (control) group. On the contrary, the metastases in the αCTLA-4 group were less evident and the same applies to the αCTLA-4 + PDT group. In order to quantify these results, we defined a constant ROI with the area corresponding to the anatomical location of the lungs in all animals and determined the radiance of the lungs in each animal at various timepoints (Fig. 6B).

The radiance of the control and αPD-1 groups increases approximately exponentially with time, which is the expected development in the absence of a therapeutic response. PDT transitorily reduces the radiance from the metastasis, but after day 17 they grow again. ANOVA with a TukeyHSD post-hoc test using time as an independent variable gives a statistically significant increase in metastasis from days 4 to 17 in the αCTLA4 and αCTLA4 + PDT groups, but not in the αCTLA4 + PDT group from days 4 to 23. This means that metastases in this group at day 23 are no longer different from metastases at day 4, although they were different between days 4 and 17. The αCTLA4 group maintains at day 24 a significant difference in metastasis with respect to day 4.

The metastases growth rates were analysed with the Gompertz sigmoid function^46^

$$S\left( t \right) \, = S_{\max } \exp \left\{ {{-}\exp \left[ {{-}k_{G} \left( {t{-}T_{i} } \right)} \right]} \right\}$$

where *S*(*t*) is the size of the tumour (in our case, radiance of metastases), *S*_max_ = *S*(*t* $\to$ ∞) is the asymptotic limit of tumour size, *k*_G_ is a growth-rate coefficient and *T*_i_ is the time at the inflection point. We linearized this function

$$\ln [\ln \left( {S_{\max } } \right){-}\ln \left( {S\left( t \right)} \right] \, = k_{G} T_{{\text{i}}} {-}k_{{\text{G}}} t$$

to calculate the growth-rate coefficient given the theoretical metastases limit. Considering the highest radiance measure in the ROI, ~ 10^11^ p/s/cm^2^/sr, we set *S*_max_ to 7 × 10^12^ p/s/cm^2^/sr. Values of *S*_max_ within a factor 5 of the selected value have a small impact on the trends of exhibited by *k*_G_. Supplementary Fig. S4 show that redaporfin-PDT changes the kinetics of metastases 12 days post-PDT from growth to remission, but the metastases regrow again after day 17. Figure 6C shows that metastases regress (negative growth coefficient) after day 17 in αCTLA-4 and αCTLA-4 + PDT groups, and the growth rate of the αCTLA-4 + PDT groups is statistically significant relative to control (*p* < 0.05). Unfortunately, some primary tumours arrived at the humanitarian endpoint after day 23.

The high expression of CD80 and low expression of CTLA-4 by 4T1 cells post-PDT raised the expectation that the combination αCTLA-4 + PDT would show therapeutic benefit. The significant reduction in lung metastases in this combination suggests benefit but survival was compromised by the poor response of the primary tumour. This motivated the evaluation of redaporfin infiltration in tumours using PAT.

### Tumour priming with photoacoustic waves followed by PDT

The rapid proliferation of 4T1-luc2 tumours in the mammary gland may be an example of growth-induced solid stress^47^, which forces blood vessels apart, reduces vascular density, compresses blood and lymphatic vessels, limits the delivery of oxygen and hinder the delivery of therapeutics. The difficulty of anticancer drugs to access all of the cells in the TME is recognized in chemotherapy^48^ and can be expected to be a more limiting factor for larger molecules and immunotherapies.

**Figure 7:**
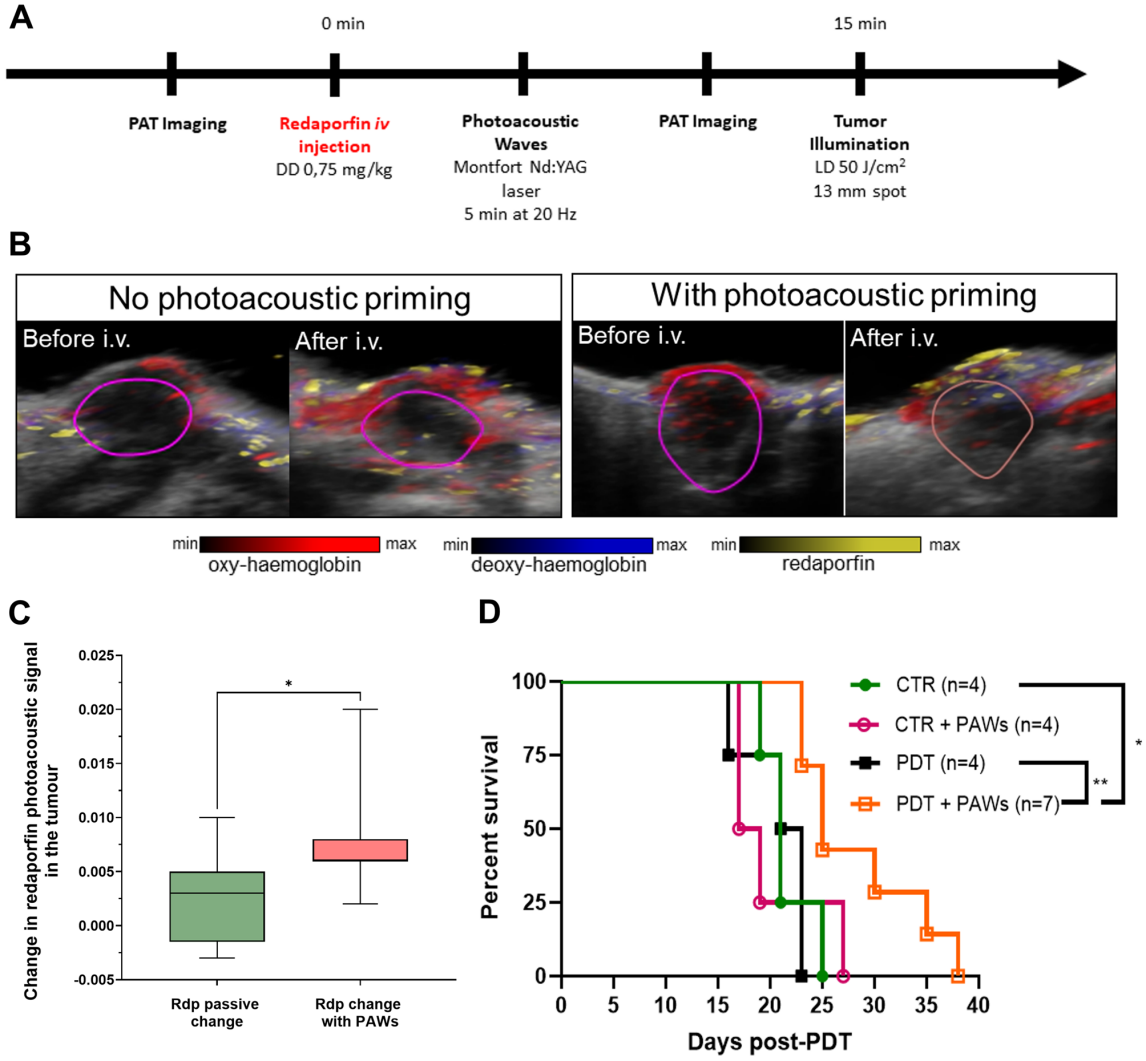
Kaplan–Meier curve representing % survival of tumour bearing mice. The animals were subjected to tumour priming with photoacoustic waves (PAWs) and followed by vascular-PDT with redaporfin, using frontal illumination. (**A**) Protocol of vascular-PDT, photoacoustic priming of the tumours and PAT imaging. (**B**) Representative PAT images of tumours before and after administration of redaporfin, in the absence or presence of photoacoustic priming. (**C**) Relative changes in the photoacoustic signal assigned to redaporfin in the tumour, before (background signal) and 10 min after administration of redaporfin without or with exposure to photoacoustic waves. (**D**) Survival of BALB/c mice with orthotopic 4T1 tumours subject to vascular-PDT and, in one group, exposure to photoacoustic waves. The exposure lasted for 5 min using nanosecond laser pulses at 20 Hz and materials that generate peak pressures of ~ 60 bar.

Figure 7A presents the protocol for imaging 4T1 tumours with PAT prior to the administration of redaporfin, followed by tumour priming with photoacoustic waves (PAWs) and then imaging again with PAT. This procedure allowed for the quantification of redaporfin in the tumour after photoacoustic priming and its comparison without priming, Fig. 7B. Priming increased the concentration of redaporfin in the tumours by a factor of 3 relative to i.v. administration alone^49^, Fig. 7C. The median survival increased from 17 days in the untreated group to 21 days in the redaporfin-PDT group (Fig. 7D), but this increase was not statistically significant. However, tumour priming with photoacoustic waves followed by vascular-PDT with redaporfin further increased the median survival to 25 days and led to a statistically significant increase in survival (Fig. 7D).

The use of PAT at 750 nm with ~ 20 mJ/cm^2^ pulses at 20 Hz after administration of redaporfin generates sub-therapeutic ROS doses. It is known that low-dose PDT can relieve drug delivery barriers in solid tumours, increase intra-tumoral drug accumulation and improve treatment response^50^. However, we did not observe changes in the response to ICB in animals subject to PAT imaging. The ROS produced under our imaging conditions are below the photodynamic priming threshold. The increased response to PDT after exposure to photoacoustic waves can be assigned to TME remodelling by 6000 pressure pulses of ~ 60 bar peak compressional pressures. Safe and transient permeabilization of biological barriers with such pressure pulses has been demonstrated and assigned to pressure gradients of 5 bar/µm that exert strong mechanical forces at the cellular level^51,52^. Such micromechanical forces facilitate tumour infiltration, effectively priming the TME.

## Discussion

To the best of our knowledge, this is the first report showing that PDT stimulates tumour cells to display high levels of CD80 on their surface. Such large CD80 expressions enables CD28 co-stimulation by increased CD28:CD80 interactions and subsequent antitumour T cell immunity.

Redaporfin-PDT also increases the expression of CTLA-4 in B16F10 and 4T1 cells, but to a much lower level than CD80. This increase in CTLA-4 may compete with CTLA-4 in lymphocytes for αCTLA-4 and less mAbs will be available to block *trans*-CTLA-4:CD80 interactions. Increased CTLA-4 post-PDT may be offset by the much higher increase in CD80. CT26 cells do not overexpress CTLA-4 after PDT. The remarkably increase in the survival of mice with CT26 tumours treated with the combination of αCTLA-4 with redaporfin-PDT can be assigned to the high surface expression of CD80 and unchanged expression of CTLA-4 post-PDT. The same principles may apply to the combination of αCTLA-4 with Bremachlorin-PDT^53^.

The concurrent expression of CD80 and PD-L1 on the surface of the same tumour cells will promote *cis*-CD80:PD-L1 interactions. Increased *cis*-PD-L1:CD80 binding in tumour cells has the potential to disrupt the *trans*-PD-1:PD-L1 binding responsible for T-cell anergy, but will not disrupt *trans*-CD80:CD28 binding and its costimulatory effect^32,39^. The increase in the expression of PD-L1 by tumour cells after padeliporfin-PDT was suggested to increase αPD-1 blockade of the PD-1:PD-L1 axis^54^. Indeed, PD-L1 expression is required for response to αPD-L1 and this biomarker predicts clinical response to ICB therapies^55^. However, excess PD-L1 expression post-PDT may demand higher αPD-L1 doses and exhaust ICBs. We propose that successful αPD-L1 + PDT combinations will depend on the relative extent of CD80 versus PD-L1 expression post-PDT. If the overexpression of CD80 is higher than of PD-L1, then the αPD-L1 + PDT combination may present therapeutic advantages. Our work shows that these overexpressions depend on the nature of the tumour (Fig. 2E,F).

The relatively low overexpression of PD-L1 and CTLA-4 on the surface of 4T1 cells after PDT, and the high overexpression of CD80, raised the expectation that PDT-ICB combinations could improve the treatment outcome in this tumour model. Orthotopic 4T1 tumours are notoriously difficult to treat with PDT because vital organs are located just underneath the tumour, which limits the light dose that can be delivered without side effects. Although the αCTLA-4 + PDT combination led to significant remission of lung metastases of orthotopic 4T1 tumours, it did not increase the survival. Using PAT, we identified an additional factor present in the primary tumour that impeded the success of this therapeutic combination: lowered drug infiltration. It is interesting to note that the phototoxicity of redaporfin-PDT in vitro increases in the order CT26 < B16F10 < 4T1, but this order is inversed in the response of the corresponding tumours to redaporfin-PDT in vivo. This is consistent with the observation that access to the tumour cells in vivo, i.e., the TME, is a critical factor to the success of the therapy.

PAT offers a detailed view of tumour vasculature and blood oxygen saturation before and after PDT, based on oxy- and deoxy-haemoglobin imaging^56–58^. Oxy- and deoxy-haemoglobin signals indicate that orthotopic 4T1 tumours have less oxyhaemoglobin than the subcutaneous tumours studied in this work. Moreover, imaging of redaporfin distribution in tumours revealed that it is mostly found in the periphery of orthotopic 4T1 tumours, suggesting a role for solid stress and elevated interstitial fluid pressure in these tumours. High peak-pressure (~ 60 bar) photoacoustic waves with central frequencies ~ 20 MHz correspond to changes of 50 bar in 10 ns or, considering the speed of sound propagation in tissues, a change of 50 bar in 15 µm. Dramatic pressure changes are produced on the scale of the size of a cell. They do not elicit adverse effects in normal tissues, but the mechanical forces exerted by such pressure pulses in the TME drive micromechanical priming of solid tumours. This micromechanical priming enabled redaporfin-PDT to significantly increase the survival of BALB/c mice bearing orthotopic 4T1 tumours.

This work uses changes in immune cell expression on the surface of tumour cells after PDT and photosensitizer mapping in tumours with PAT, to provide a simple rational for the combination of PDT with ICB therapies. Redaporfin-PDT stimulates the expression of CD80 in CT26, B16F10 and 4T1 tumour cells and makes them more immunogenic. Some of the effects expected from the overexpression of CD80 is dampened by the overexpression of CTLA-4 in B16F10 and 4T1 cells and by overexpression of PD-L1 in CT26 and B16F10 cells. The combination αCTLA-4 + PDT was particularly successful to cure mice bearing subcutaneous CT26 tumours and to reduce lung metastases of mice with orthotopic 4T1 tumours. It was particularly challenging to reduce the burden of the primary orthotopic 4T1 tumours because tumour biophysics restrict the access of oxygen and drug inside these tumours. However, micromechanical priming with photoacoustic waves improved the response to redaporfin-PDT in this challenging tumour model.

### Outlook

Fighting cancer requires multidisciplinary approaches. We combined ICBs with a photosensitizer and light to improve the therapeutic outcome of diverse tumour models, and guided these therapies with bioluminescence and photoacoustic imaging. Our data supports a mechanism where CD80 is overexpressed by tumours cells after PDT, and sometimes also PD-L1 and CTLA-4. Favourable combinations of PDT with αPD-L1 or with αCTLA-4 are expected for high CD80/PD-L1 or CD80/CTLA-4 overexpression ratios. However, solid tumour biophysics may restrict drug infiltration and render increased immunogenicity ineffective to promote immunotherapy. This is the case of orthotopic 4T1 tumours, which require micromechanical priming to respond to therapeutics. Knowledge of the overexpression of immune molecules on the surface of tumour cells after PDT together with photoacoustic imaging of tumours, inform on the combinations between photodynamic and immune therapies that are most likely to offer clinical benefits.

## Materials and methods

### Cell culture

CT26 cells (ATCC Cat# CRL-2638), 4T1 cells (ATCC Cat# CRL-2539), 4T1-luc2 cells (Caliper Life Sciences) and B16F10 (offered by IPO, Porto, Portugal) were cultured in Dulbecco’s Modified Eagle’s medium supplemented with 10% heat-inactivated foetal bovine serum, 100 U/ml penicillin and 100 ng/ml streptomycin.

### In vitro PDT

5 × 10^4^ cells of CT26, B16F10 and 4T1 were seeded in 24-well plates and left overnight in the incubator to attach. In order to evaluate the expression of immune molecules by tumour cells after PDT in vitro, redaporfin (Luzitin, Portugal) was added from a stock dimethyl sulfoxide solution to obtain 40–60% cell viability for a light dose of 0.3 J/cm^2^ at 740 nm. We found that, under these conditions, the redaporfin concentrations that led to the desired viability were: 0.20 µM for CT26, 0.05 µM for B16F10 and 0.02 µM for 4T1. These conditions exposed the cells to a significant PDT effect while leaving a large number of cells alive to evaluate the expression of immune molecules. After 24 h of incubation with these concentrations of redaporfin, the cells were washed twice, fresh medium was added, and then illuminated. Six hours later the cells were collected and analysed by flow cytometry.

### Flow cytometry and expression of immune checkpoint molecules

The following anti-mouse mAbs were used for flow cytometry surface staining: Brilliant Violet 650™ anti-mouse CD80 (BioLegend), APC anti-mouse CD152 (CTLA-4) (BioLegend) and Brilliant Violet 605™ anti-mouse CD274 (B7-H1, PD-L1) (BioLegend). Zombie Violet™ Fixable Viability Kit (BioLegend) was used to assess the cell viability. Cells were stained with the viability kit at room temperature for 20 min, washed and re-suspended in FACS buffer containing the antibodies for the surface staining (30 min at 4 °C). Cells were then washed twice with FACS buffer, re-suspended in this buffer, and analysed by flow cytometry using a Flow Cytometer NovoCyte 3000.

### Mouse models and treatments

The animal experiments were approved by the Portuguese Animal Health Authority (DGAV authorization 0421/000/000/2020, carried out in accordance with relevant guidelines and regulations, and in compliance with the ARRIVE guidelines.). CT26 and B16F10 tumours were established by s.c. injection of 3.5 × 10^5^ CT26 or 5 × 10^5^ B16F10 cells in Matrigel:PBS (1:1) in the right flank of BALB/cAnNCrl or C57BL/6NCrl mice, respectively. 4T1-luc2 tumours were established by orthotopical injection of 2 × 10^4^ cells in the right abdominal mammary fat pad of BALB/cAnNCrl mice. The animals (Charles River Laboratories) were ~ 10 weeks old (~ 20 g) when the tumours were established. PDT was performed when the tumour largest diameter was $\phi$ > 5 mm and animals were randomly assigned to each group. The implementation of the 4T1-luc2 tumour model ensured that lung metastases could occur even if surgical resection of the whole tumour was performed in the day of the treatment (Supplementary Figure S3)^59^. Vascular-PDT of s.c. CT26 tumours with diameters $\phi$ = 5 mm achieved 87% cure rate with 0.75 mg/kg of redaporfin and a light dose of 50 J/cm^2^^19^. To investigate possible benefits of ICB + PDT combinations, we lowered the dose (0.6 mg/kg) or treated larger tumours ($\phi$ = 6 mm), which are subtherapeutic conditions with no cures. Redaporfin-PDT led to complete remission of all subcutaneous B16F10 tumours with 1.5 mg/kg and 74 J/cm^2^ at DLI = 15 min with a Pluronic P123 formulation^18^. At these doses, lethality is observed with the Kolliphor EL formulation in clinical use^17^ employed in our studies. We decreased redaporfin dose to 1 mg/kg to obtain a 10% cure rate without lethality. The location of orthotopic 4T1 tumours in mice makes vascular-PDT difficult to perform without damage to underlying tissues. We tested longer DLIs (Supplementary Table S1) and different geometries for the illumination (Supplementary Figure S5), and opted for DLI = 72 h with 1.8 mg/kg of redaporfin and 100 J/cm^2^ in transversal illumination. A diode laser at 748 nm (Omicron, Germany) was employed. The endpoint was defined as a tumour diameter of 10–12 mm, or least 60 days of tumour-free survival post-PDT. mAbs were administered in the following dosages: anti-mouse CTLA-4 (CD152, clone UC10-4F10-11, BioXCell) 5 mg/kg, anti-mouse PD-1 (CD279, clone RMP1-14, BioXCell) 12.5 mg/kg, mouse isotype control (IgG2b, Clone MPC-11, BioXCell) 5 mg/kg. Redaporfin was formulated with < 1% Kolliphor EL^17,60^.

### Bioluminescence imaging

4T1-luc2 cells express luciferase. Lung metastases were visualized with luminescence imaging (IVIS Lumina XR, Caliper LifeSciences, USA) 7 min post-i.p. administration of 150 mg/kg of D-luciferin in PBS. A depilatory cream was applied in the mice thorax to remove hair prior to the experiments. Mice were anesthetized immediately after i.p. administration, using isoflurane (1.5–2.5%) and kept under anaesthesia to prevent motion during acquisition. Bioluminescent signals were quantified with the Living Image software (RRID:SCR_014247) and are expressed as radiant efficiency (p/s/cm^2^/sr)/(μW/cm^2^). A region-of-interest (ROI) was drawn in the lung area of each animal and maintained for comparison over time and between treatment groups.

### Photoacoustic tomography (PAT)

Redaporfin accumulation on tumour was assessed using PAT (Vevo LAZR-X imaging system, 40 MHz transducer, Fujifilm/VisualSonics, Canada). Anaesthesia was induced and maintained using isoflurane (1.5–2.5%) while imaging. Each animal was placed in a favourable position for acquiring the image (supine for 4T1 and prone for CT26 and B16F10), the tumour region was covered with gel for ultrasound, the transducer was positioned normal to the centre of the tumour, and scanning was initialized. Molecular images were acquired in the PA-mode between 680 and 970 nm with 5 nm steps, and B-mode ultrasound was used to collect anatomical images. Photoacoustic spectra were acquired in a fixed position, one axial slice, varying the wavelength from 680 to 970 nm. 3D multiwavelength (MW) acquisition was performed by scanning the whole tumour at specific wavelengths (680, 740, 750, 765, 924 and 966 nm) and with a 3D step size of 0.5 mm. Gain values of B- and PA-mode were set at the beginning and maintained for the whole experiment. Unmixing of the MW data was performed with the Vevo LAB software (FUJIFILM/VisualSonics) and using the PA spectra of redaporfin, oxy- and deoxy-haemoglobin (Supplementary Figure S6). A measure of redaporfin in the tumour was obtained with the following procedure: (1) the photoacoustic signal of redaporfin at each point in the tumour was extracted from the signal collected at 6 wavelengths from 680 to 970 nm; (ii) the signal assigned to redaporfin was integrated over all the tumour and the background signal, with the same components and in the same volume before the administration of redaporfin, was subtracted; (iii) this is done for all animals (before and after for each one of them) and averaged for the group of animals without exposure to PA waves and for the group of animals with exposure to such waves. Real time accumulation of redaporfin in the tumour (2D section of the tumour centre) was followed by PAT with single excitation at 750 nm to allow real time recording (Supplementary Figure S1). Time zero was set as the time immediately before intravenous administration of redaporfin and the accumulation in the tumour was followed for a few minutes.

### Tumour priming with photoacoustic waves

Photoacoustic waves with mostly compressive broadband pressure peaks of ~ 60 bar were generated by the absorption of 8 ns laser pulses (M-NANO Nd:YAG Montfort Laser) by light-to-pressure transducers made of carbon nanoparticles and polydimethylsiloxane (LaserLeap Technologies, Portugal)^61^, using ~ 60 mJ/cm^2^ laser fluences. Such pressure pulses are known to permeabilize biological barriers^51,52^. A layer of acoustic coupling gel was placed over the epilated tumour, the light-to-pressure transducer was pressed against the gel and the tumour was exposed to photoacoustic waves for 5 min at 20 Hz.

### Statistical analysis

Statistical analyses were performed using GraphPad Prism or KaleidaGraph. The results are presented as the mean ± standard error of the mean (SEM). One-Way ANOVA with Dunnett’s (GraphPad Prism) or TukeyHSD (KaleidaGraph) post-tests were used to determine statistically significant differences of the means between control and treated groups. Survival analysis employed a Kaplan–Meier estimator. Statistical differences are presented at probability levels *p < 0.05, **p < 0.01, ***p < 0.001 and ****p < 0.0001.

## Supplementary Information

Supplementary Information.

Supplementary Video 1.

Supplementary Video 2.

Supplementary Video 3.

Supplementary Video 4.

## Data Availability

The datasets used and/or analysed during the current study available from the corresponding author on reasonable request.
