# Supplementary material for: Photodynamic therapy changes tumour immunogenicity and promotes immune-checkpoint blockade response, particularly when combined with micromechanical priming
Source: Sci Rep. 2023 Jul 19;13:11667. doi: 10.1038/s41598-023-38862-8 (PMC10356828; doi:10.1038/s41598-023-38862-8)
Supplement: Supplementary file 1 — Supplementary Information. [file 41598_2023_38862_MOESM1_ESM.pdf]

Photodynamic Therapy Changes Tumour Immunogenicity and Promotes Immune-Checkpoint Blockade Response, Particularly When Combined with Micromechanical Priming

Catarina S. Lobo, M. Inês P. Mendes, Diogo A. Pereira, Lígia C. Gomes-da-Silva*, Luis G. Arnaut*

CQC, Chemistry Department, University of Coimbra, 3004-535 Coimbra, Portugal

Supplementary data

Figure S1. 2D PAT of redaporfin in CT26 tumors using excitation at 750 nm. Frames of real-time PAT **(A)** before i.v. administration. **(B)** 10 sec after administration. **(C)** 30 sec after administration. **(D)** photoacoustic spectra of the tumor before and 15 min after redaporfin administration.

Figure S2. Kinetics of tumor volume growth for PDT and immunotherapies in three different tumor models.

Figure S3. Exploratory studies with the orthotopic 4T1 tumor model to determine the treatment day when tumor resection does not prevent lung metastases.

Table S1. Redaporfin-PDT parameters of the several tested protocols for 4T1-luc2 mammary carcinoma model.

Table S2. Macroscopic evaluation score criteria for PDT treatment impact on primary tumors.

Figure S4. Expression of the irradiance of 4T1-luc2 metastases in the lungs according to the linearized Gompertz function.

Figure S5. Schematic representation of frontal (conventional) **(A)** and transversal **(B)** illumination of 4T1-luc2 tumors.

Figure S6. Normalized photoacoustic spectra of redaporfin, oxy- and deoxy-hemoglobin.

Video S1. Real-time tracking of redaporfin tumor accumulation assessed by PAT imaging of the CT26 tumor region (8x speed up). The red marker on the timeline represents the i.v. injection time.

Video S2-S4. 3D-rendered photoacoustic/ultrasounds representation of CT26 (S2), B16F10 (S3) and 4T1-luc2 (S4) tumors, after intravenous redaporfin administration. The timepoints of acquisition are specified for each case.

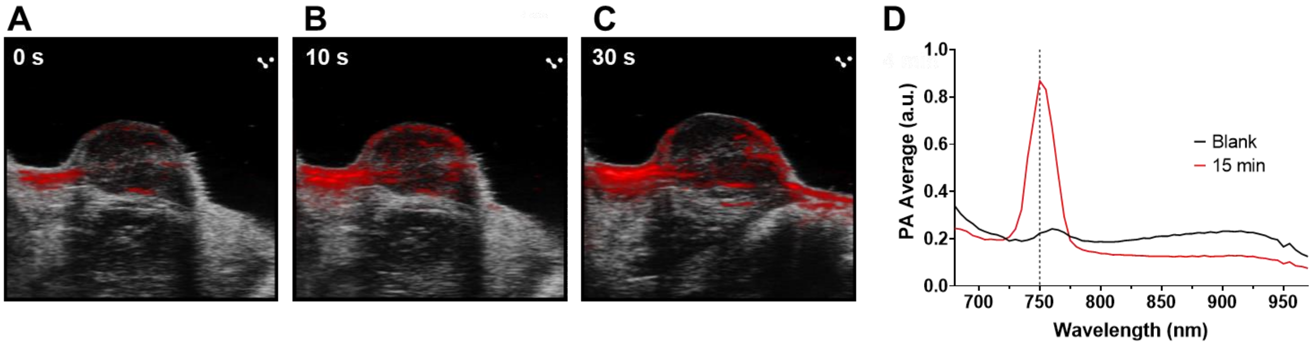

Figure S1. 2D PAT of redaporfin in CT26 tumors using excitation at 750 nm. Frames of real-time PAT **(A)** before i.v. administration. **(B)** 10 sec after administration. **(C)** 30 sec after administration. **(D)** photoacoustic spectra of the tumor before and 15 min after redaporfin administration.

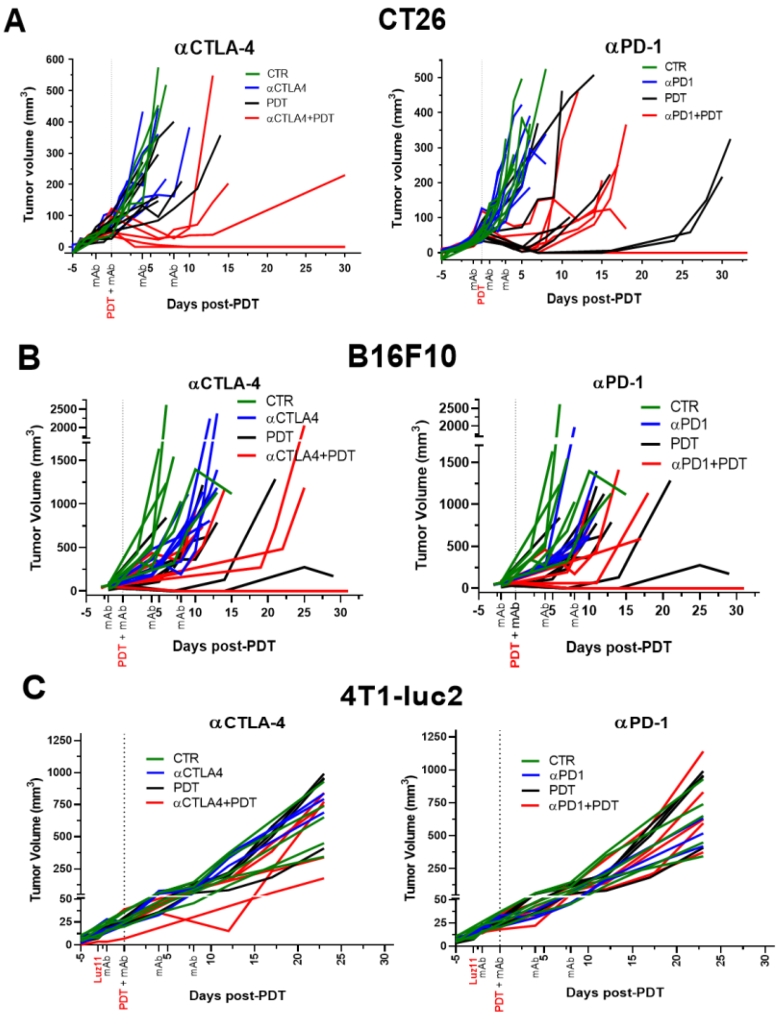

Figure S2. Kinetics of tumor volume growth for PDT and immunotherapies in three different tumor models. Tumor volumes of **(A)** CT26, **(B)** B16F10 and **(C)** 4T1-luc2 models are represented individually for each animal with the colors corresponding to the treatment group. Schedule of administrations of immunotherapy antibodies are indicated in the x-axis, as well as the PDT treatment day, which was established as the day 0 of the experiment.

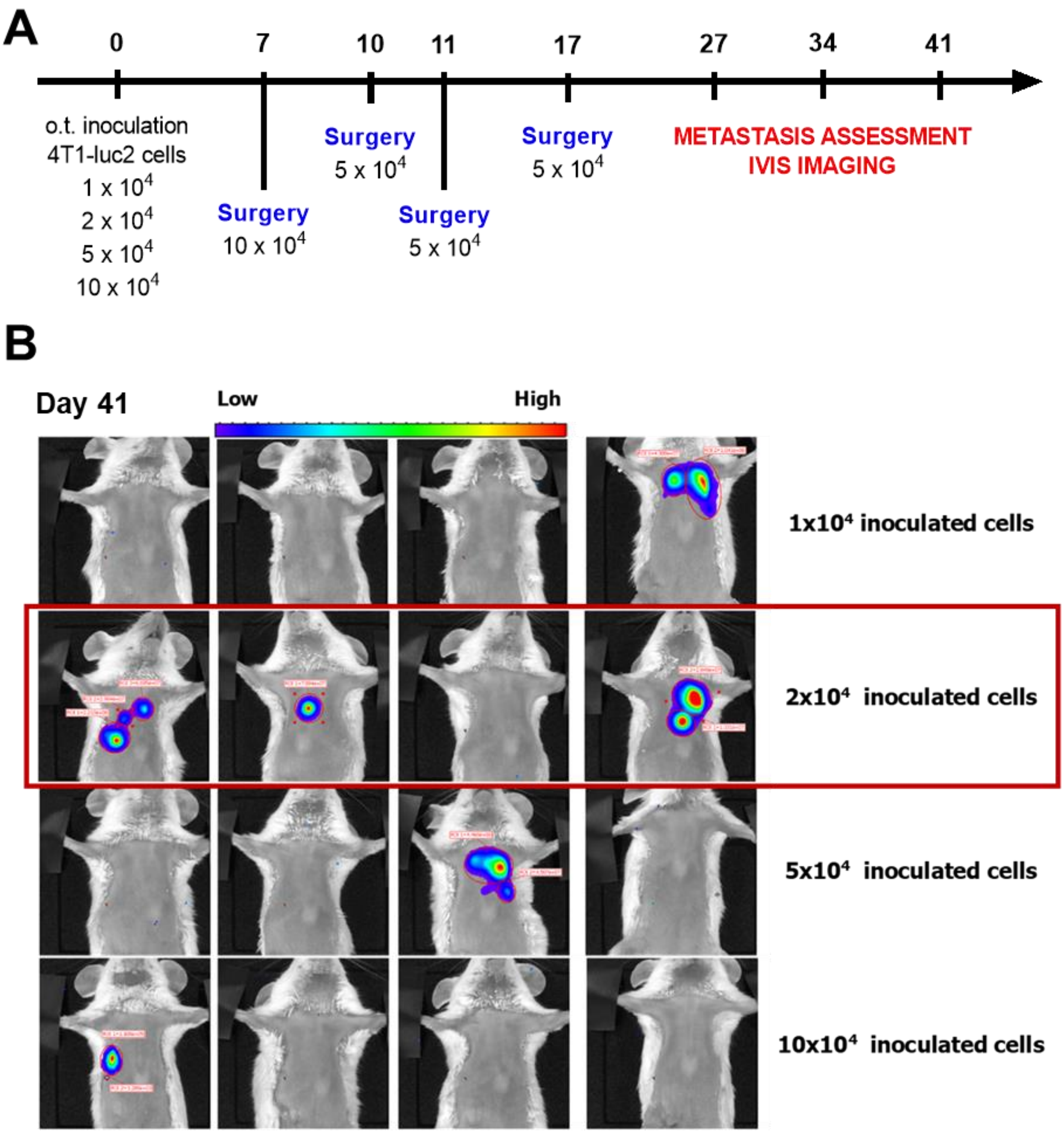

Figure S3. Exploratory studies with the orthotopic 4T1 tumor model to determine the treatment day when tumor resection does not prevent lung metastases. **(A)** Schematic outline of metastatic model optimization protocol: Different number of cells were inoculated in the mammary gland ($1x10^4$, $2x10^4$, $5x10^4$ and $10x10^4$ cells) and surgery was performed when tumor diameter attained 4-5 mm. Bioluminescence imaging was performed on days 27, 34 and 41. **(B)** IVIS bioluminescence of 4T1-luc2 mammary carcinoma tumor at day 41, showing that the inoculation of $2x10^4$ cells originated the highest metastasization.

Table S1. Redaporfin-PDT parameters of the several tested protocols for 4T1-luc2 mammary carcinoma model. Drug and light doses, illumination spot diameter and fluence were tested to obtain the best outcome in terms of impact on treatment. Macroscopic alterations on tumor, as edema and necrosis, were evaluated and scored according to criteria on Table S2. o.t. - orthotopic; s.c. - subcutaneous; Prtcl - Protocol: V - vascular protocol; C - cellular protocol; CV - cellular and vascular protocol; TV/TC - transversal irradiation of tumor, as schematized in Figure S2, with vascular/cellular protocol.

Prtcl	Model	Drug dose (mg/kg)	DLI	Light dose (J/cm^2)	Spot diameter (cm)	Fluence (mW/cm^2)	Survival to PDT	Impact Score	
								Edema	Necrosis
**V1**	o.t.	1.00	15 min	50	1.3	130	43 %	**0**	**1**
**V2**	o.t.	0.75	15 min	50	1.3	130	100 %	**0**	**1**
**V3**	o.t.	0.75	15 min	50	1.1	182	100 %	**0**	**1**
**V4**	o.t.	1.00	15 min	50	1.1	182	100 %	**0**	**1**
**V5**	s.c.	1.00	15 min	50	1.3	130	100 %	**2**	**0**
**V6**	s.c.	1.00	15 min	100	1.3	130	100 %	**2**	**1**
**C1**	o.t.	1.00	72 h	100	1.3	130	100 %	**0**	**2**
**C2**	o.t.	2.00	72 h	50	1.0	220	100 %	**2**	**1**
**C3**	o.t.	2.00	72 h	75	1.0	131	0 %	**1**	**1**
**C4**	o.t.	2.00	72 h	65	1.0	131	0 %	**1**	**1**
**C5**	o.t.	1.00	72 h	100	1.3	130	100 %	**1**	**1**
**C6**	o.t.	1.00	72 h	120	1.0	150	83 %	**0**	**1**
**C7**	o.t.	1.00	48 h	120	1.0	150	0 %	**0**	**1**
**C8**	o.t.	1.00	96 h	120	1.0	150	100 %	**0**	**1**
**CV1**	o.t.	1.00<br>0.5	72 h<br>15 min	80	1.3	130	0 %	**-**	**-**
**CV2**	o.t.	1.00<br>0.5	72 h<br>15 min	60	1.3	130	100 %	**1**	**1**
**CV3**	o.t.	1.00<br>0.5	24 h<br>15 min	50	1.0	220	0 %	**-**	**-**
**CV4**	o.t.	1.00<br>1.00<br>0.4	144 h<br>72 h<br>15 min	50	1.0	220	100 %	**1**	**1**
**CV5**	o.t.	2.00<br>0.4	72 h<br>15 min	50	1.0	220	0 %	**-**	**-**
**TV1**	o.t.	1.8	15 min	120	1.1	178	100 %	**1**	**1**
**TC1**	o.t.	1.0	48 h	120	1.1	178	100 %	**1**	**1**
**TC2**	**o.t.**	**1.8**	**72 h**	**100**	**1.2**	**137**	100 %	**1**	**2**
**TC3**	o.t.	1.8	72 h	150	1.2	137	100 %	**1**	**2**

Table S2. Macroscopic evaluation score criteria for PDT treatment impact on primary tumors.

**Edema** increased blood vessel wall permeability due to inflammation and obstruction of fluid clearance in the lymphatic system		**Necrosis** black necrotic tissue formed when healthy tissue dies and becomes dehydrated, typically because of local ischemia	
no edema	**0**	no necrosis	**0**
light edema	**1**	mild erythema	**1**
strong edema	**2**	superficial necrosis	**2**
		deep necrosis	**3**

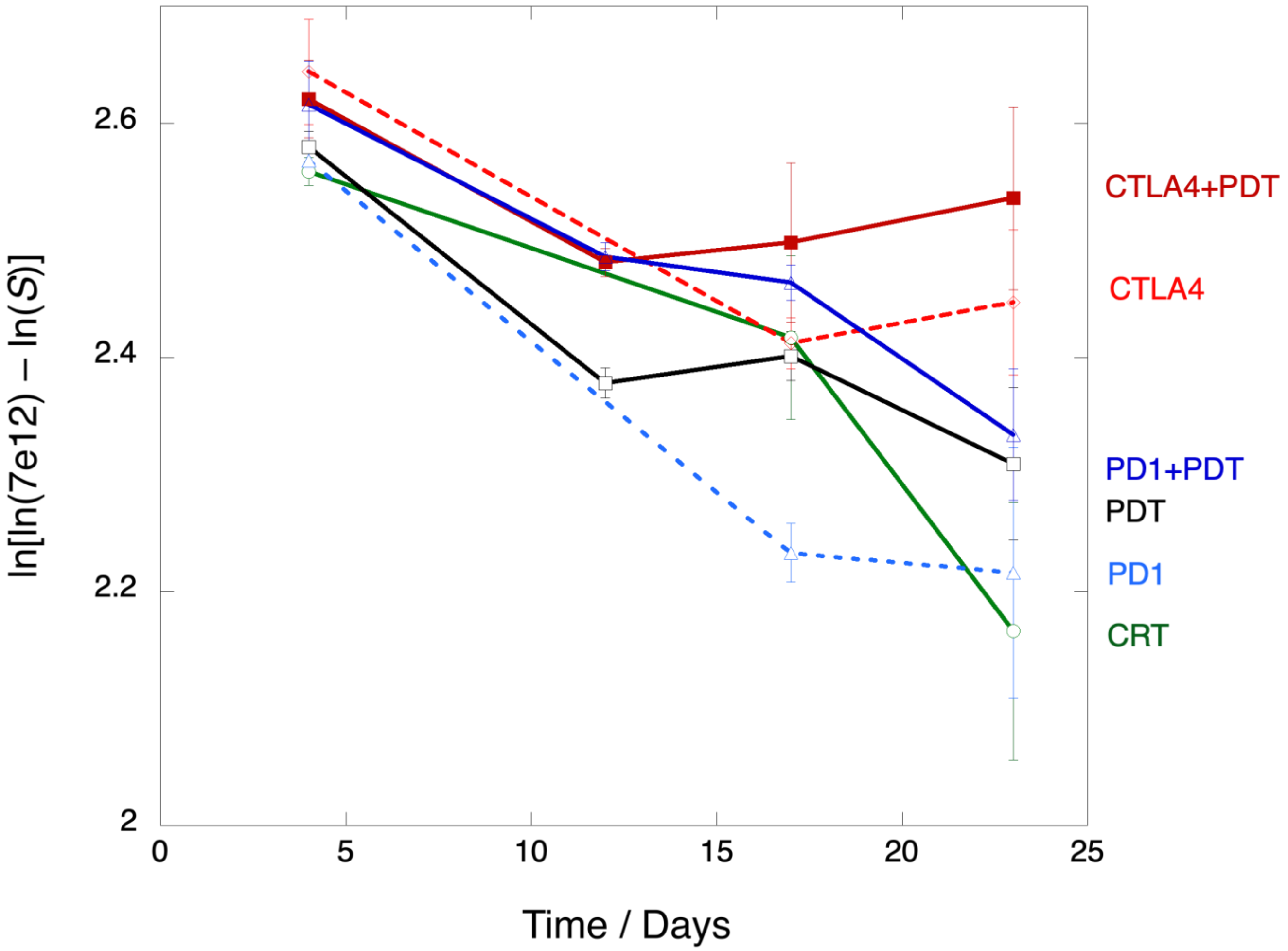

Figure S4. Expression of the irradiance of 4T1-luc2 metastases in the lungs according to the linearized Gompertz function. The PDT group changes temporarily the sign of the growth kinetics, but the αCTLA-4 and αCTLA-4+PDT groups invert definitively the kinetics, from growth to remission.

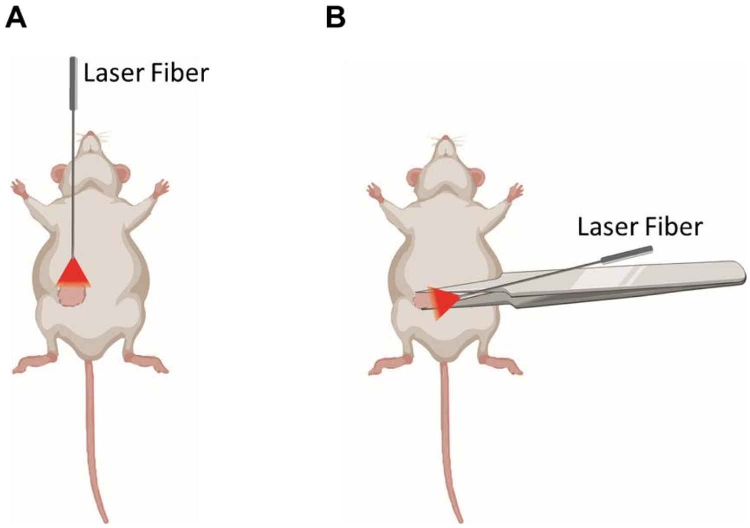

Figure S5. Schematic representation of **(A)** frontal (conventional) and **(B)** transversal irradiation of 4T1-luc2 tumors.

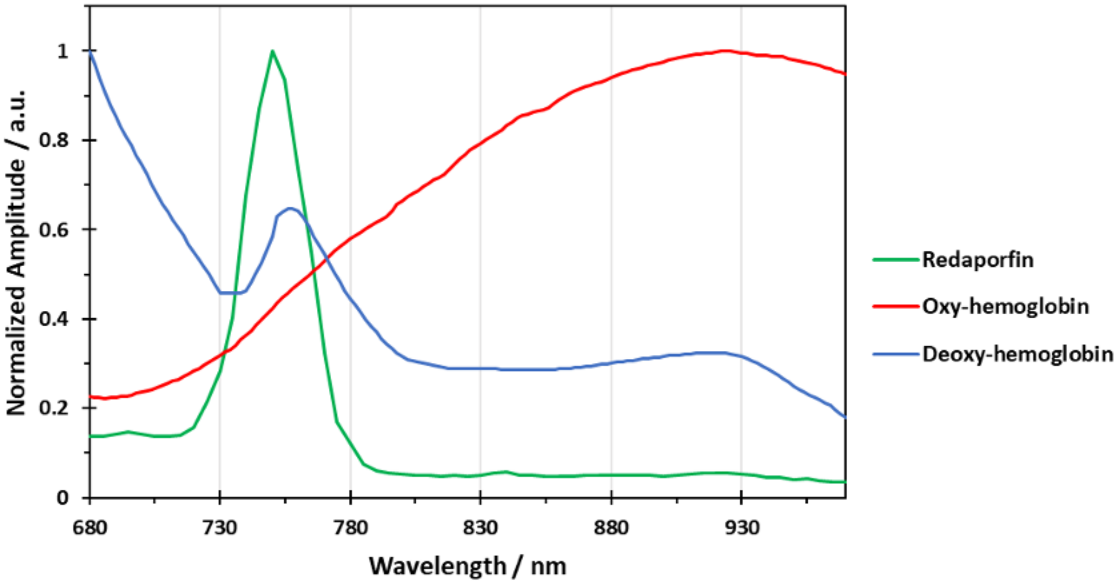

Figure S6. Normalized photoacoustic spectra of redaporfin, oxy- and deoxy-hemoglobin. Redaporfin was prepared in Kolliphor EL formulation and its photoacoustic spectrum was acquired in a phantom apparatus with the Vevo LAZR-X multimodal imaging system. Oxy- and deoxy-hemoglobin photoacoustic spectra are part of the Vevo LAZR-X analysis software.
